# Oncogenic LINC00698 suppresses apoptosis of melanoma stem cells to promote tumorigenesis via LINC00698-miR-3132-TCF7/hnRNPM axis

**DOI:** 10.1186/s12935-024-03408-z

**Published:** 2024-07-28

**Authors:** Anas Mohammed, Ahmad Khan, Xiaobo Zhang

**Affiliations:** College of Life Sciences, Laboratory for Marine Biology and Biotechnology of Pilot National Laboratory for Marine Science and Technology (Qingdao), Southern Marine Science and Engineering Guangdong Laboratory (Zhuhai), Zhejiang University, Hangzhou, 310058 People’s Republic of China

**Keywords:** Melanoma stem cells, LncRNA, Apoptosis, Stemness, Tumorigenesis

## Abstract

Melanoma progression depends on melanoma stem cells (MSCs), which are distinguished by the distinct dysregulated genes. As the key factors in the dysregulation of genes, long non-coding RNAs (lncRNAs) take great effects on MSCs. However, the underlying mechanism of lncRNAs in MSCs has not been extensively characterized. To address the roles of lncRNAs in MSCs, LINC00698 was characterized in this study. The results revealed that LINC00698 was upregulated in MSCs, showing its important role in MSCs. The further data indicated that the LINC00698 silencing triggered cell cycle arrest in the G0/G1 phase and apoptosis of MSCs. LINC00698 could directly interact with miR-3132 to upregulate the expression of TCF7, which was required for sustaining the stemness and the tumorigenic potency of MSCs. At the same time, LINC00698 could bind to the hnRNPM protein to enhance the protein stability, thus suppressing apoptosis and promoting the stemness of MSCs. Furthermore, the in vivo data demonstrated that LINC00698 was essential for tumorigenesis of MSCs via the LINC00698-miR-3132-TCF7/hnRNPM axis. Therefore, our findings contributed novel insights into the underlying mechanism of melanoma progression.

**Supplementary Information:**

The online version contains supplementary material available at 10.1186/s12935-024-03408-z.

## Introduction

Melanoma is a malignant melanocyte tumor that typically develops in the skin. The prevalence of melanoma in men and women has increased over the past three decades [1]. As reported, tumorigenesis of melanoma depends on melanoma stem cells [2]. The solid tumor is composed of a diverse cell population that differs in morphology, function and molecular signatures [3]. In this cell population, cancer stem cells, having similar physiological features to typical stem cells, such as self-renewal and differentiation, are essential for tumor initiation, immunoevasion, progression, metastasis and drug resistance [4]. Thus, melanoma stem cells (MSCs) are the most promising targets for cancer therapy to reduce the risk of cancer recurrence [5]. MSCs, the key cell population causing melanoma metastasis and disease progression [6, 7], are distinguished by their distinct dysregulated genes [8]. More and more evidences indicate that non-coding RNAs play essential roles in the dysregulation of genes [9–11]. Non-coding RNAs, including microRNAs (miRNAs) and long non-coding RNAs (lncRNAs), can regulate the characteristics and stemness of cancer stem cells via targeting the key genes of cells [12, 13]. In recent decades, the functional significance of non-coding RNAs in cancers as well as cancer stem cells has become particularly apparent for miRNAs [2, 14, 15]. In melanoma, various mechanisms mediated by miRNAs have been employed to control the properties of MSCs [2, 14, 15]. However, the roles of lncRNAs in cancers and cancer stem cells have not been extensively explored.

LncRNAs, the non-coding RNAs of more than 200 nucleotides in length, involve in many biological processes, such as epigenetic regulation, cell proliferation, cell differentiation and apoptosis [16, 17]. The cumulative data have revealed that lncRNAs play essential roles in the regulation of gene expression through controlling transcription, post-transcription and histone modification via interaction with other RNAs or proteins [18–20]. In MSCs, lncRNAs can regulate tumorigenesis through different biological mechanisms [21, 22]. Some lncRNAs contain miRNA response elements, thus acting as competitive endogenous RNAs (ceRNAs) via sponging the desired miRNAs to regulate the expression of their target genes [23]. LINC01291 promotes melanoma aggressiveness by acting as a ceRNA that sponges miR-625-5p to sustain the expression of insulin-like growth factor 1 receptor (IGF-1R) [24]. Another important regulatory role of lncRNAs includes the lncRNA-protein interactions via the RNA-binding domains of proteins, which may contribute to tumor growth and progression [25, 26]. SAMMSON, a lncRNA that promotes melanoma cell viability by influencing mitochondrial activity and translation processes, modulates the localization of CARF, an RNA-binding protein that sequesters the exo-ribonuclease XRN2 [27]. In MSCs, LHFPL3-AS1-long, an isoform of LHFPL3-AS1, is interacted with polypyrimidine tract binding protein 1 (PTBP1) to mediate alternative splicing, which is required for maintaining the stemness of MSCs [28]. Although the accumulated evidence has shown that lncRNAs are involved in MSCs, the underlying mechanism of lncRNAs in MSCs has not been well characterized.

In order to explore the involvement of lncRNAs in melanoma stem cells (MSCs), the upregulated lncRNAs in MSCs were evaluated in this study. Based on the lncRNA sequencing data [28], LINC00698 was significantly upregulated in MSCs, suggesting that LINC00698 mediated a regulatory mechanism in MSCs. Therefore, the molecular mechanism of LINC00698 in sustaining the activity of MSCs was further investigated. The results revealed that LINC00698 was essential for the maintenance of tumorigenicity of MSCs via regulating TCF7 expression by sponging miR-3132, offering novel insights into the pathogenesis of melanoma.

## Materials and methods

### Cell culture

Melanoma stem cells (MSCs) were sorted from cell lines MDA-MB-435 and A375 previously in our laboratory [15]. MSCs were cultured at 37 °C in a humidified atmosphere with 5% CO_2_ in a serum-free Dulbecco’s modified Eagle’s/F12 (DMEM/F-12) medium (Gibco, USA) supplemented with 20 ng/mL epidermal growth factor (Beyotime, China), 10 ng/mL basic fibroblast growth factor (Beyotime, China), 5 µg/mL of insulin (Beyotime, China) and 2% of B-27 (Sigma, USA). MDA-MB-435 was cultured in Leibovitz’s L-15 medium (Gibco, USA) supplemented with 10% fetal bovine serum (FBS), while A375 was cultured in DMEM (Gibco, USA) supplemented with 10% FBS. Both cell lines were cultured at 37 °C in a 100% humidified atmosphere. They were purchased from The Cell Bank of The Chinese Academy of Sciences (Shanghai, China).

### Sequencing and expression analysis of lncRNAs

Total RNAs from MSCs or melanoma non-stem cells (MNSCs) were isolated using Trizol (Generay Biotech, China) following the manufacturer’s instructions. The ribosomal RNA was eliminated by the Ribo-Zero™ kit (Epicentre, Madison, WI, USA). The fragmented RNAs were subjected to the first-strand and the second strand cDNA synthesis. Then. the adaptor ligation and enrichment were performed using a low-cycle method according to the instructions provided by NEBNext^®^ Ultra™ RNA Library Prep Kit for Illumina (New England Biolabs Incorporation, USA). The refined library products were assessed using the Agilent 2200 TapeStation and Qubit^®^2.0 (Life Technologies, USA). The libraries were subjected to the paired-end sequencing by Guangzhou RiboBio Co., Ltd. (Guangzhou, China) using the IlluminaHiSeq 3000 platform.

The differential expression analysis of lncRNAs in MSCs and MNSCs was performed with the DESeq2 package (v1.20.0). The differentially expressed lncRNAs were allocated based on fold change > 2 and *p* < 0.05. Following the identification of the significantly differentially expressed genes, heatmap graphical representation was constructed on the normalized gene expression data using GraphPad Prism Software version 8.0.1 (GraphPad Software, San Diego, USA).

### Quantitative real-time PCR

Total RNAs were extracted from cells with RNA Isolation Kit (Generay Biotech, China). The cDNA was reversely transcribed from the total RNAs using HiScript II Q RT SuperMix for qPCR (Vazyme Biotech Co, China). The quantitative real-time PCR reaction mixture (10 µl) contained RNase-free ddH_2_O, 5 µl of SYBR Master Mix, 0.2 µl ROX dye 1, 0.2µl of forward primer, and 0.2 reverse primer mixed with the template. The primers were sequence-specific (LINC00698, 5’-ATCACGTAGCTGGTTGCTGG-3’ and 5’-GGCAAGTCTGCTACTCGTCA-3’; ALDH1, 5’-GATGCCGACTTGGACA ATGC-3’ and 5’-TCTTAGCCCGCTCAACACTC-3’; Sox2, 5’-AACCAGCGCATGGA CAGTTA-3’ and 5’-GACTTGACCACCGAACCCAT-3’; Nanog, 5’-AATGGTGTGAC GCAGGGATG-3’ and 5’-CTATAGCCAGAGACGGCAGC-3’; Oct4, 5’-TGTCAGGGC TCTTTGTCCAC-3’ and 5’-TCTCCCCAGCTTGCTTTGAG-3’; GAPDH, 5’-AATGGG CAGCCGTTAGGAA-3’ and 5’-GCGCCCAATACGACCAAATC-3’; miR-3132, 5’-AA TGGTGTGACGCAGGGATG-3’ and 5’-CTATAGCCAGAGACGGCAGC-3’; U6, 5’-C TCGCTTCGGCAGCACA-3’ and 5’-AACGCTTCACGAATTTGCGT-3’; BCL6, 5’-AC CTCCCACTCCCATGTGTC-3’ and 5’-TTGTTCTCCACCACCTCACG-3; PAX6, 5’-C TTCGCTAATGGGCCAGTGA-3’ and 5’-TCACTCCGCTGTGACTGTTC-3’; TCF7, 5’ -TTAAGGAGAGCGCTGCCATC-3’ and 5’-CCAGTTTGTCTCTGTGGTGGAT-3’; hnRNPM, 5′-GAGCAATGCAAAAGGCTGGA-3′ and 5′-ATCAATGGGTTGCCCTCC TG-3′).

### Data mining

The expression profile of LINC00698, TCF7 or hnRNPM in cancerous tissues was retrieved from The Cancer Genome Atlas (TCGA) database (https://cancergenome.nih.gov/). Tissue samples included two groups (cancerous tissues and normal tissues). Kaplan–Meier survival analysis of clinical cases was collected and evaluated by gene expression profile interactive analysis (GEPIA) (http://gepia.cancer-pku.cn/index.html).

### Gene silencing and rescue and miRNA overexpression

To knock down the expression of TCF7 or LINC00698 in cells, the short hairpin RNA (shRNA) targeting a gene (TCF7-shRNA, 5’-TGGTAATGGACAAGAGTCACT-3’; LINC00698-shRNA, 5’-GCACTAGCTGTGAACACTAAG-3’) was cloned into the lentiviral vector pLent-U6-Puro (GenePharma, Shanghai, China). As a control, the scrambled shRNA (shRNA-scrambled, 5’-TTCTCCGAACGTGTCACGT-3’) was also cloned into pLent-U6-Puro. A shRNA was transfected into HEK293T cells using Lipofectamine 2000 (Life Technologies, USA). Forty-eight hours later, the packaged virus was collected to infect melanoma stem cells. After screening with puromycin (10 µg/ml), the cells stably expressing the target shRNA were obtained. Subsequently the cells were subjected to examine the gene silencing.

To silence the expression of hnRNPM in MSCs, RNA interference (RNAi) assay was carried out. MSCs (1 × 10^6^) were transfected with 50 nM of a sequence-specific siRNA (hnRNPM-siRNA, 5’-GGCAUAGGAUUUGGAAUAATT-3’, siRNA-scrambled, 5’-UUCUCCGAACGUGUCACGUTT-3’) using Lipofectamine 2000 (Life Technologies, California, USA). All the siRNAs were synthesized by GenePharma Co., Ltd. (Shanghai, China). At different time after transfection, the cells were harvested for later use.

To rescue the TCF7 expression in the TCF7-silenced cells, the TCF7 gene was mutated to avoid the recognition by TCF7-shRNA at nucleotide positions 767 and 773 from T to C and A to G, respectively. The TCF7-mutated sequence was amplified using the sequence-specific primers (5’-ATGTACAAAGAGACCGTCTACTCC-3’ and 5’- GA CAGTGACTCCTGTCCGTTACC-3’; 5’-CTTCCTCTAGCCCAGCTTGA-3’ and 5’-GG TCTCAAACTACTGACGTCA-3’) and cloned into pcDNA3.1^+^ vector (Promega, USA). Subsequently, TCF7-shRNA and the recombinant plasmid were co-transfected into MSCs with Lipofectamine 2000 (Life Technologies). The endogenous TCF7 was silenced by TCF7-shRNA, while the recombinant plasmid could express the recombinant TCF7 protein. At different time after transfection, the cells were collected for further use.

For miRNA overexpression, the synthesized miR-3132 (5’-UGGGUAGAGAAGGA GCUCAGAGGA-3’) at 50 nM was transfected into cells using Lipofectamine 2000 (Life Technologies, USA) according to the manufacturer’s instructions. As a control, the control miRNA (5’-AUCCUACGACAGUGCCGGAGAAU-3’) was included in the transfection. The miRNAs were synthesized by GenePharma (Shanghai, China).

### Cell proliferation assay

To evaluate the cell proliferation, MTS [3-(4, 5-dimethylthiazol-2-yl)-5-(3 carboxymethoxyphenyl)-2-(4-sulfophenyl)-2 H-tetrazolium, inner salt] of CellTiter 96^®^ Aqueous One Solution Cell Proliferation Assay kit (Promega, USA) was used in accordance with the manufacturer’s instructions. In brief, cells were plated in 96-well plates (5 × 10^3^ cells/well) and then 20 µl of CellTiter 96^®^ AQueous One Solution Reagent was added to each well. After incubation at 37 °C in a humidified atmosphere for 2 h, the absorbance was estimated at 490 nm using the iMARKTM microplate reader (Bio-Rad, USA).

### Cell cycle assay

Fluorescence-activated cell sorting (FACS) analysis was used to examine the cell cycle. Cells were centrifuged at 300×*g* for 10 min. After washes with phosphate buffered saline (PBS), the cells were incubated with propidium iodide (PI) (Sigma, USA) and 200 µg/ mL of RNaseA (Sangon, China) for 30 min at 37 °C in the dark. Subsequently, the cells were examined using a FACS-Calibur flow cytometer (BD Biosciences, USA). Flow cytometric data were analyzed using Cell Quest Pro software (BD Biosciences, USA).

### Analysis of caspase 3/7 activity

The caspase 3/7 activity of cells was evaluated using the Caspase-Glo 3/7 assay (Promega, USA) according to the manufacturer’s protocol. Cells at a density of 1 × 10^4^ cells/well were plated in a 96-well plate and added with 100 µl of caspase-Glo 3/7 reagent (Promega, USA). After incubation in the dark at room temperature for 30 min, the luminescence of the cells was measured.

### Apoptosis detection with Annexin V

Fluorescein isothiocyanate (FITC)-annexin V apoptosis detection kit (BD Biosciences, USA) was used to test apoptosis according to the manufacturer’s protocol. Briefly, cells were resuspended in binding buffer at 1 × 10^6^ cells/ml. Then the cells were stained with FITC-annexin V and propidium iodide (PI). The mixture was incubated in the dark at room temperature for 15 min. After washes with PBS, the cells were analyzed by flow cytometry (CyteFLEX, Beckman Coulter, USA).

### Western blot analysis

Cells were lysed in RIPA lysis buffer (Beyotime Institute of Biotechnology, Shanghai, China) with 2 mM phenylmethanesulfonyl fluoride (PMSF, Solarbio, Beijing, China) on ice. The proteins of the cell lysates were separated on a 10% sodium dodecyl sulfate-polyacrylamide gel electrophoresis (SDS-PAGE) and then electro-transferred to a nitrocellulose membrane (GE Healthcare, Waukesha, WI, USA) in transferring buffer (25 mM Tris-HCl, 190 mM glycine, 20% methanol, pH8.0). After blocking with 5% non-fat milk in TBST buffer (20 mM Tris-HCl, 150 mM NaCl, 0.05% Tween-20, pH 8.0) for 60 min at room temperature, the membrane was incubated with a primary antibody (Abcam, USA) in TBST buffer containing 1% non-fat milk overnight at 4^0^C. Subsequently, the membrane was incubated with the horseradish peroxidase (HRP)-conjugated secondary antibody (BioRad, Hercules, CA, USA) for 3 h at room temperature. The membrane’s signal was acquired using a 5-bromo-4-chloro-3-indolyl phosphate/nitro blue tetrazolium substrate (Sangon, China).

### Tumorsphere formation assay

Tumorsphere formation assay was performed using an ultralow adhesion plate and serum-free cell culture growth medium. Cells, seeded at a density of a single cell into an ultralow adherent 96-well plate (Corning, USA), were cultured for 14 days at 37 °C with 5% CO_2_. For miRNA and siRNA assays, 50 nM of a miRNA mimic or siRNA was transfected into the cells using Lipofectamine 2000 (Thermo Fisher, USA) every 3 days. The spheres with a diameter greater than or equal to 50 μm were counted under a light microscope.

### Prediction of target miRNAs of lncRNA and target genes of miRNAs

The prediction of the target miRNAs of lncRNA was conducted using the online algorithmic tools including miRDB (http://www.mirdb.org/), miRanda (http://www.microrna.org/) and RNA22 v2.0 (https://cm.jefferson.edu/rna22/). The overlapped miRNAs were considered the possible targets of lncRNA.

Three algorithms, including TargetScan (https://www.targetscan.org/), starBase (https://starbase.sysu.edu.cn) and miRTarBase (https://mirtarbase.cuhk.edu.cn/), were used to predict the target genes of miRNAs. The overlapped genes were the potential targets of miRNAs.

### Dual-luciferase reporter assay

LINC00698 was cloned into the luciferase reporter vector pmirGLO (Promega, USA) using LINC00698-specific primers (5’-CTCGAGCTGCACTTTGAGGATGCA-3’ and 5’-TCTAGAGCAAGTGCTTGGAACAGACA-3’). As a control, LINC00698 was mutated and then cloned into pmirGLO using sequence-specific primers (5’-TTGAGCTC TTCAAGCAGTGGAGA-3’ and 5’-TCTAGAAATCCCATCTTCCTCTCC-3’). The recombinant plasmid and one of the synthesized miRNAs (miR-346, miR-3132 and miR-185-3p) were co-transfected into MSCs using Lipofectamine 2000 (Thermo Fisher, USA). At 48 h after transfection, the firefly and renilla luciferase activities were measured using the dual luciferase reporter assay system (Promega, USA) according to the manufacturer’s protocol.

### RNA pull-down assay

To explore the direct interaction between LINC00698 and miR-3132, MSCs were transfected with 50 nM of biotin-labeled LINC00698 (5’-CTCTTTTAATGTTCTA ATG TTTTCT-biotin-TEG-3’) (biotin-TEG, biotin with 15 atom triethylene glycol spacer). As a control, the scrambled sequence of LINC00698 was also labeled with biotin (biotin-control, 5’-ATGCAGCTGGAGAGGAAGATGGGA-biotin-TEG-3’) and transfected into MSCs. The biotin-labeled lncRNAs were synthesized by Youkang Biotechnology Inc (Hangzhou, China). At 48 h after transfection, the cells were lysed by cell lysis buffer (Beyotime Biotechnology, Shanghai, China) for 20 min, followed by centrifuge at 14,000×g for 15 min. Then, the cell lysate was incubated with the streptavidin magnetic beads (Beyotime Biotechnology, Shanghai, China) for 2 h at 4℃ with gentil rotation. After washes with cell lysis buffer, the magnetic beads were subjected to extract miRNAs.

To screen the proteins bound to LINC00698, the synthesized biotin-labeled LINC00698 (Youkang Biotechnology Inc.) was coupled to the streptavidin magnetic beads (Beyotime Biotechnology, Shanghai, China) in binding and washing buffer II (10mM Tris-HCl, 1mM EDTA, 2 M NaCl, 0.05% Tween-20, pH 7.5) for 30 min at room temperature. At the same time, the cells were lysed with cell lysis buffer (Beyotime Biotechnology, Shanghai, China). Subsequently, the lncRNA-conjugated beads were incubated with the cell lysate for 2 h at 4℃. After washes of the beads with cell lysis buffer containing PMSF (Solarbio, Beijing, China), the pulled down proteins were eluted using 0.1% SDS, followed by examination of proteins using SDS-PAGE. The proteins were identified using mass spectrometry.

### Electrophoretic mobility shift assay (EMSA)

EMSA was conducted to examine the interaction between lncRNA and protein. A lncRNA (10 µM) was incubated with serial concentrations of the recombinant protein in reaction buffer (0.1 M KCl, 1 mM dithiothreitol, 1 mM MgCl_2_, 10 mM 4-(2- hydroxyethyl)-1-piperazineethanesulfonic acid, pH7.60 at 37 °C for 30 min. Subsequently, the mixture was separated by 1% agarose gel electrophoresis at 100 V for 30 min, followed by staining with ethidium bromide to detect RNA bands.

### Prediction of lncRNA secondary structure

To predict the lncRNA secondary structure, the lncRNA sequence was analyzed by the online RNAfold tool (http://rna.tbi.univie.ac.at/cgi-bin/RNAWebSuite/RNAfold. cgi). The stable stem-loop structure of the RNA secondary structure was obtained.

### Tumorigenicity assay in vivo

MSCs stably expressing LINC00698-shRNA or shRNA-scrambled were resuspended in physiological saline and then mixed with Matrigel (Corning, USA) at a ratio of 1:1. The cell suspension (collected 6 × 10^5^) were subcutaneously inoculated into 5-week-old female non-obese diabetes/severe combined immunodeficiency (NOD/SCID) mice. The tumor volume was examined every five days. Seven weeks later, the mice were euthanized and the intact solid tumors were taken out. Fresh tissues from xenograft tumors were immediately flash-frozen in liquid nitrogen and kept at − 80 °C for further experiments. All the animal experiments were performed according to the protocols approved by The China Institutional Animal Care and Use Committee (IACUC) of Zhejiang University (Ethical approval No. 19,792).

### Immunohistochemistry assay

The solid tumors of mice were cut into Sect. (5 μm-thick) and then placed on a precoated slide with 3-triethoxysilylpropylamine (Merck, Darmstadt, Germany). The slide was immersed in xylol for 1 h and successively treated with alcohol at a series of decreasing concentrations. After deparaffinising the section, antigen retrieval of the tissue was performed in a microwave for 5 min in TEC buffer (0.05 M Tris-HCl, 0.05 M ethylenediaminetetraacetic acid, 0.02 M sodium citrate, pH7.8), followed by peroxidase blocking. Subsequently the slide was incubated with a primary antibody overnight at 4 °C. After washing with PBS, a subsequent incubation with the biotinylated secondary antibody (Vector, Grünberg, Germany) was performed for 30 min. The slide was stained with diaminobenzidine (Sigma, USA) for 10 min to label proteins and then counterstained with hematoxylin to label nuclei.

### Protein stability assay

Protein stability was assessed by cycloheximide chase assays as described previously [29]. Briefly, MSCs were transfected with a vector expressing LINC00698 or vector alone as a control. At 36 h after transfection, the cells were treated with 50 µg/ml cycloheximide (Sigma-Aldrich, Massachusetts, USA) to inhibit the protein synthesis. The cycloheximide-treated cells were harvested at different time points (0, 2, 4 and 6 h) and then subjected to Western blotting. β-actin was used as an internal control.

### Statistical analysis

All numeral data were indicated as mean ± standard deviation (mean ± SD). Statistical significance between treatments were evaluated with Student’s t-test and one-way analysis of variance (ANOVA). Each experiment was conducted with three distinct biological replicates.

## Results

### Upregulation of LINC00698 in melanoma stem cells and melanoma tissues

**Fig. 1:**
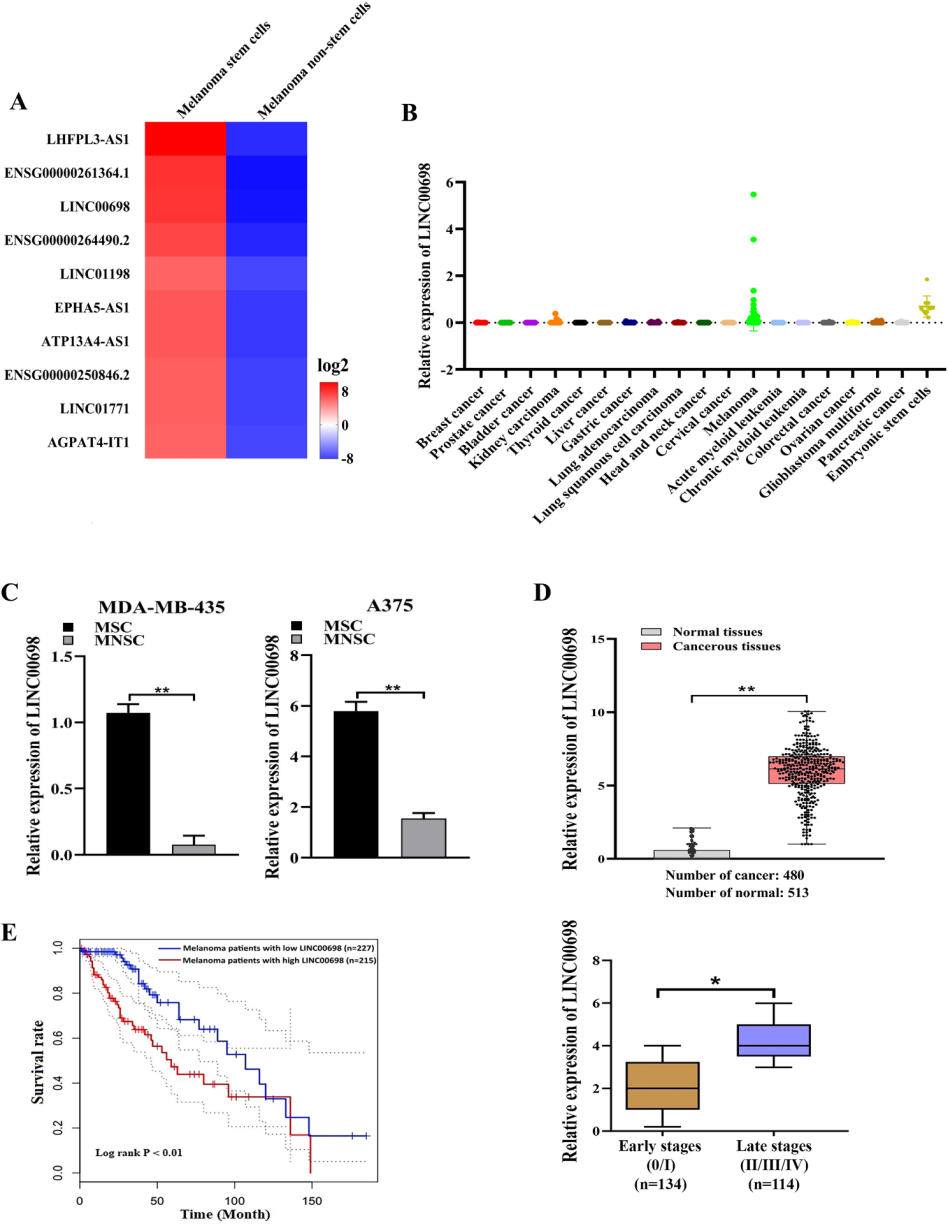
Upregulation of LINC00698 in melanoma stem cells and melanoma tissues. (**A**) Upregulation of lncRNAs in melanoma stem cells (MSCs). The top 10 upregulated lncRNAs in MSCs were shown. (**B**) Expression profile of LINC00698 in various cancers. Based on the MiTranscriptome database, the expression level of LINC00698 in various cancers was characterized. (**C**) The differential expression of LINC00698 in MSCs and MNSCs. The differential expression level of LINC00698 was examined using quantitative real-time PCR (**, *p* < 0.01). (**D**) The expression level of LINC00698 in melanoma patients. The expression levels of LINC00698 in cancerous tissues and non-cancerous normal tissues of melanoma patients as well as at the early stages (0/I) and late stages (II/III/IV) of melanoma were characterized based on the TCGA database ((*, *p* < 0.05; **, *p* < 0.01). (**E**) Survival scheme of melanoma patients with low vs. high expression of LINC00698

To explore the involvement of lncRNAs in melanoma stem cells (MSCs), the upregulated lncRNAs in MSCs were characterized. Based on the lncRNA sequencing data available in our laboratory [28], top 10 lncRNAs were upregulated in MSCs compared with melanoma non-stem cells (MNSCs) (Fig. 1A)  (Supplementary Material 2). Among them, LINC00698 was significantly upregulated in melanoma based on the MiTranscriptome database (Fig. 1B and Fig S1). Thus, LINC00698 was further characterized in MSCs.

The results showed that LINC00698 was significantly upregulated in MSCs compared with MNSCs (Fig. 1C), suggesting that LINC00698 was associated with melanoma tumorigenesis.

To evaluate LINC00698 in clinic, the expression level of LINC00698 in patients with melanoma was characterized based on the TCGA database. The results demonstrated that LINC00698 was significantly upregulated in cancerous tissues compared to normal tissues and at the late stage of melanoma (Fig. 1D). At the same time, the Kaplan–Meier survival rate analysis revealed that the patients with higher LINC00698 expression had worse overall survival than those with lower LINC00698 expression (Fig. 1E). These data indicated that LINC00698 was upregulated in melanoma patients and associated with the disease progression.

Collectively, these findings revealed that LINC00698 was upregulated in MSCs, suggesting its important role in melanoma progression.

### Promotion of tumorigenicity of melanoma stem cells by LINC00698

**Fig. 2:**
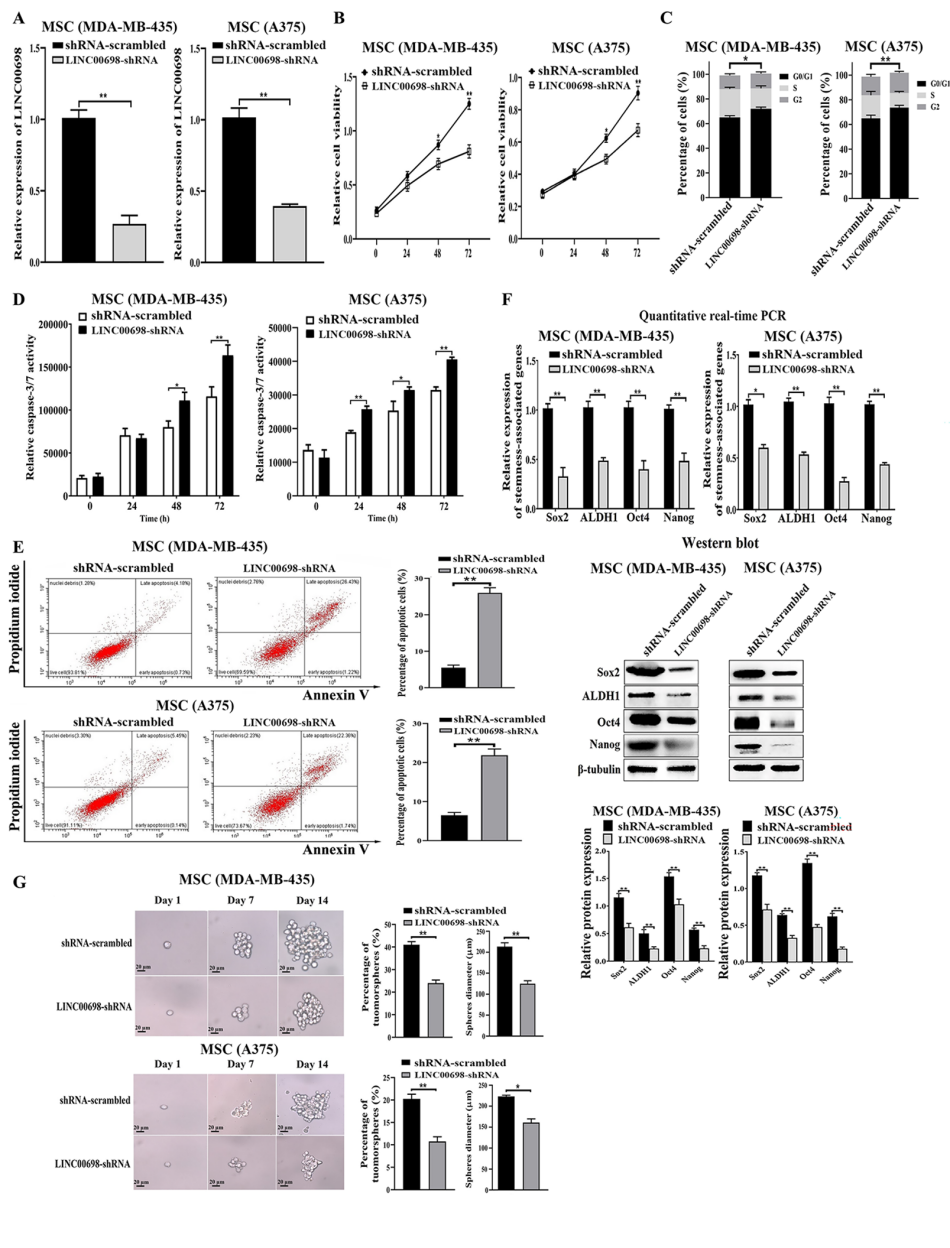
Promotion of tumorigenicity of melanoma stem cells by LINC00698. (**A**) Silencing of LINC00698 in MSCs. MSCs (MDA-MB-435 and A375) were transfected with LINC00698-shRNA or shRNA-scrambled to stably express a shRNA. The LINC00698 expression level was examined using quantitative real-time PCR (**, *p* < 0.01). (**B**) Impact of LINC00698 silencing on the viability of MSCs. MSCs (MDA-MB-435 and A375) were transfected with LINC00698-shRNA. Then the cell viability was examined (*, *p* < 0.05; **, *p* < 0.01). (**C**) Influence of LINC00698 silencing on cell cycle. MSCs (MDA-MB-435 and A375) were treated with shRNA-scrambled or LINC00698-shRNA, followed by the examination of cell cycle (*, *p* < 0.05; **, *p* < 0.01). (**D**) Effects of LINC00698 silencing on apoptosis of MSCs. MSCs (MDA-MB-435 and A375) were transfected with LINC00698-shRNA or shRNA-scrambled and then the caspase 3/7 activity of cells was evaluated (*, *p* < 0.05; **, *p* < 0.01). (**E**) Evaluation of apoptosis of MSCs with flow cytometry. The LINC00698-shRNA-transfected MSCs (MDA-MB-435 and A375) were subjected to apoptosis detection using Annexin V (**, *p* < 0.01). The shRNA-scrambled was used as a control. (**F**) Downregulation of stemness genes in LINC00698-silenced MSCs. The MSCs transfected with LINC00698-shRNA were subjected to quantitative real-time PCR and Western blot to examine the expressions of stemness factors (Sox2, ALDH1, Nanog and Oct4) (*, *p* < 0.05; **, *p* < 0.01). The shRNA-scrambled was included in the transfection as a control. β-tubulin was used as a control (**G**) Impact of the LINC00698 silencing on tumorsphere formation of MSCs. After the transfection of MSCs (MDA-MB-435 and A375) with LINC00698-shRNA or shRNA-scrambled, the tumorsphere formation was examined every day. The percentage of tumorsphere formation was assessed (*, *p* < 0.05; **, *p* < 0.01). Scale bar, 20 μm

To reveal the roles of LINC00698 in MSCs, LINC00698 was knocked down, followed by the examinations of the properties of MSCs. Quantitative real-time PCR results displayed that the expression level of LINC00698 was significantly decreased by LINC00698-shRNA compared to the control in MSCs (Fig. 2A). The LINC00698 silencing significantly inhibited the proliferation of MSCs (MDA-MB-435 and A375) compared to the controls (Fig. 2B), indicating that LINC00698 silencing could suppress the proliferation of MSCs. Furthermore, flow cytometry analysis data showed that the percentage of cells in the G0/G1 phase after LINC00698 knockdown was significantly increased compared to the control (Fig. 2C), indicating that the LINC00698 silencing led to the cell cycle arrest of MSCs. To investigate the influence of the LINC00698 silencing on apoptosis of MSCs, the LINC00698-silenced MSCs were subjected to the examination of apoptosis. The results revealed that the LINC00698 knockdown significantly increased the caspase-3/7 activity of MSCs compared with the control (Fig. 2D). The apoptosis detection using Annexin V generated the same results (Fig. 2E). These data indicated that the LINC00698 silencing triggered apoptosis of MSCs.

To reveal the role of LINC00698 in MSCs, the expression levels of stemness-related genes of LINC00698-silenced MSCs were evaluated. The results showed that the LINC00698 silencing significantly reduced the expressions of stemness factors of MSCs (MDA-MB-435 and A375) compared with the control (Fig. 2F). The data of tumorsphere formation assays displayed that the LINC00698 silencing significantly reduced the percentage of tumorsphere formation of MSCs compared with the control (Fig. 2G).

Taken together, these data presented that the LINC00698 silencing triggered apoptosis of MSCs and suppressed the stemness of MSCs.

### Underlying mechanism of LINC00698 in MSCs

**Fig. 3:**
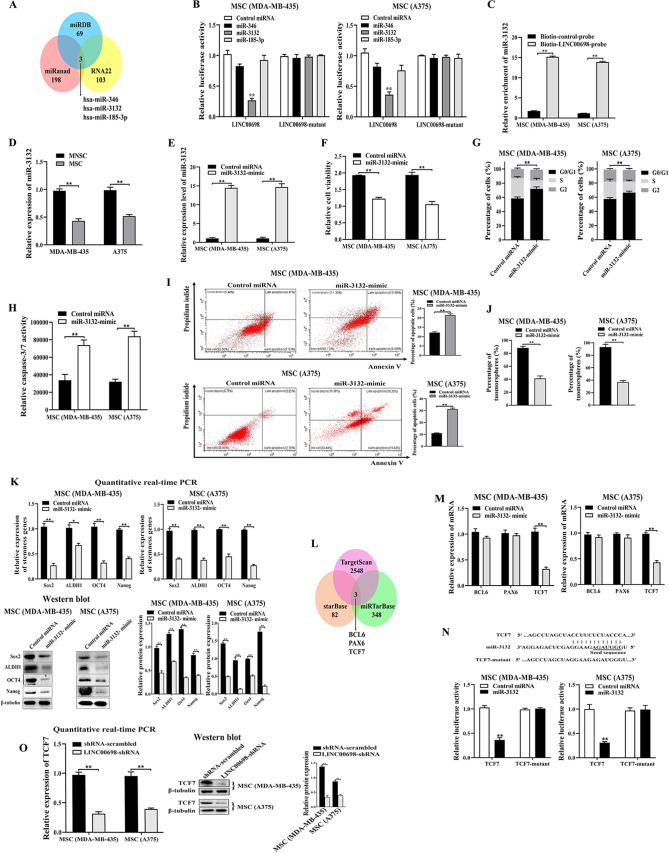
Underlying mechanism of LINC00698 in MSCs. (**A**) Prediction of target miRNAs of LINC00698. Three algorithms (miRDB, miRanda and RNA22 v2.0) were used to predict the target miRNAs of LINC00698. The overlapped miRNAs predicted by three softwares were the potential targets of LINC00698. (**B**) Direct interaction between LINC00698 and miRNAs. MSCs were co-transfected with the recombinant pmirGLO-LINC00698 plasmid and the synthesized miRNAs. As a control, pmirGLO-LINC00698-mutant was also transfected into the cells. At 48 h after trnasfection, the firefly and renilla luciferase activities were analyzed (**, *p* < 0.01). (**C**) RNA pull-down assay of LINC00698. MSCs (MBA-MD-435 and A375) were transfected with the biotinylated LINC00698 or the biotin-control. Subsequently the cell lysate was incubated with the streptavidin magnetic beads. The pulled down products were subjected to quantitative real-time PCR to detect miR-3132 (**, *p* < 0.01). (**D**) Expression level of miR-3132 in MSCs. The miR-3132 expression level in MSCs and MNSCs was detected using quantitative real-time PCR (**, *p* < 0.01). (**E**) Overexpression of miR-3132 in MSCs. MSCs (MDA-MB-435 and A375) were transfected with miR-3132 mimic or control miRNA. At 48 h after transfection, the expression level of miR-3132 was detected using quantitative real‐time PCR (**, *p* < 0.01). (**F**) Influence of miR-3132 overexpression on the viability of MSCs. The viability of miR-3132-overexpressed MSCs were examined at 48 h after transfection (**, *p* < 0.01). (**G**) Effects of miR-3132 overexpression on cell cycle. The cell cycle of mmiR-3132-overexpressed MSCs was evaluated using flow cytometry at 48 h after treatment (**, *p* < 0.01). (**H**) Effects of miR-3132 overexpression on apoptosis of MSCs. Apoptosis of the miR-3132-overexpressed MSCs (MDA-MB-435 and A375) was detected by caspase 3/7 activity assay at 48 h after transfection of miR-3132 mimic or control miRNA (**, *p* < 0.01). (**I**) Detection of apoptosis using annexin V. Apoptosis of MSCs (MDA-MB-435 and A375) was examined by flow cytometry at 48 h after transfection with miR-3132 mimic or control miRNA (**, *p* < 0.01). (J) Tumorsphere formation assay of the miR-3132-overexpressed MSCs. MSCs (MDA-MB-435 and A375) were transfected with miR-3132 mimic or control miRNA. Ten days later, the percentage of tumorsphere formation was evaluated (**, *p* < 0.01). (K) Effects of miR-3132 overexpression on the expressions of stemness genes in MSCs. The expression profiles of stemness genes in miR-3132-overexpressed MSCs were determined using quantitative real-time PCR and Western blot at 48 h after transfection (*, *p* < 0.05; **, *p* < 0.01). β-tubulin was used as a control (**L**) Prediction of target genes of miR-3132. Three algorithms (TargetScan, starBase, and miRTarBase) were used to predict the target genes of miR-3132. The overlapped genes were the potential targets of miR-3132. (**M**) Influence of miR-3132 overexpression on the expressions of predicted target genes in MSCs. The expression levels of BCL6, PAX6 and TCF7 were detected using quantitative real‐time PCR in MSCs at 48 h after transfection with miR-3132 or control miRNA (**, *p* < 0.01). (**N**) The direct interaction between miR-3132 and the target TCF7 in MSCs. MSCs were co-transfected with miR-3132 or control miRNA and TCF7 or TCF7- mutant. At 48 h after transfection, the firefly and renilla luciferase activities were analyzed (**, *p* < 0.01). (**O**) Impact of LINC00698 knockdown on the expression of TCF7 in MSCs. The LINC00698-silenced MSCs were analyzed using quantitative real-time PCR and Western blot at 48 h after treatment (**, *p* < 0.01)

To reveal the underlying mechanism of LINC00698 in MSCs, the potential target miRNAs of LINC00698 were predicted. Based on the prediction using miRDB, miRanda and RNA22 v2.0, 3 miRNAs were the potential targets of LINC00698 (Fig. 3A). To assess the direct interaction between LINC00698 and miRNAs (miR-346, miR-3132, and miR-185-3p), the dual-luciferase reporter assays were performed. The results demonstrated that miR-3132 of 3 miRNAs significantly decreased the reporter luciferase activity of MSCs transfected with pmirGLO-LINC00698 and miR-3132 compared with the control (pmirGLO-LINC00698-mutant) (Fig. 3B). However, miR-346 and miR185-3p could not significantly decreased the reporter luciferase activity of LINC00698-transfected MSCs compared with the control (Fig. 3B). These data indicated that miR-3132 was directly interacted with LINC00698. To confirm the interaction between LINC00698 and miR-3132, the RNA pull-down assay using LINC00698 was conducted. The results showed that miR-3132 was significantly enriched in the pulled down products of LINC00698 compared with the control (Fig. 3C), indicating that LINC00698 was interacted with miR-3132.

To explore the role of miR-3132 in MSCs, the expression profile of miR-3132 in MSCs and MNSCs was examined. The quantitative real-time PCR results illustrated that miR-3132 was significantly downregulated in MSCs compared with MNSCs (Fig. 3D), suggesting that miR-3132 was associated with tumorigenesis of MSCs. To assess the impact of miR-3132 on MSCs, miR-3132 was overexpressed in MSCs and then the properties of MSCs were examined. The results showed that miR-3132 was overexpressed in MSCs (Fig. 3E). The miR-3132 overexpression inhibited the proliferation of MSCs (Fig. 3F), induced the cell cycle arrest in the G0/G1 phase (Fig. 3G) and further triggered apoptosis of MSCs compared with the control (Fig. 3H and I).

To evaluate the impact of miR-3132 on the stemness of MSCs, the capacity of tumorsphere formation of miR-3132-overexpressed MSCs was examined. The results showed that the miR-3132 overexpression significantly reduced the percentage of tumorsphere formation of MSCs compared to the control (Fig. 3J). At the same time, the stemness genes were significantly downregulated in the miR-3132-overexpressed MSCs (Fig. 3K). These findings indicated that miR-3132 played a negative role in MSCs.

To explore the mechanism of miR-3132 in MSCs, the target genes of miR-3132 were characterized. As predicted, BCL6 (B cell lymphoma 6), PAX6 (paired box 6) and TCF7 (transcription factor 7) were the potential targets of miR-3132 (Fig. 3L). To confirm the target genes, miR-3132 was overexpressed in MSCs and then the expression profiles of the predicted genes were examined. The quantitative real-time PCR results showed that the miR-3132 overexpression significantly repressed the expression of TCF7 but not BCL6 and PAX6 (Fig. 3M), indicating that TCF7 was the target gene of miR-3132. To assess the direct interaction between miR-3132 and TCF7 mRNA in MSCs, the luciferase reporter assay was conducted. When the recombinant plasmid expressing TCF7 and miR-3132 were co-transfected into MSCs, the luciferase activity was significantly decreased compared with the controls (Fig. 3N), showing the direct interaction between miR-3132 and TCF7 mRNA in MSCs. To assess the influence of LINC00698 on TCF7, LINC00698 was silenced in MSCs and then the expression level of TCF7 was determined. The data of quantitative real-time PCR and Western blot analysis demonstrated that the LINC00698 silencing significantly downregulated TCF7 in MSCs (Fig. 3O), indicating that LINC00698 could promote the expression of TCF7, the target gene of miR-3132.

Collectively, these findings demonstrated that LINC00698 directly interacted with miR-3132 to upregulate the TCF7 expression.

### Requirement of TCF7 for MSCs

**Fig. 4:**
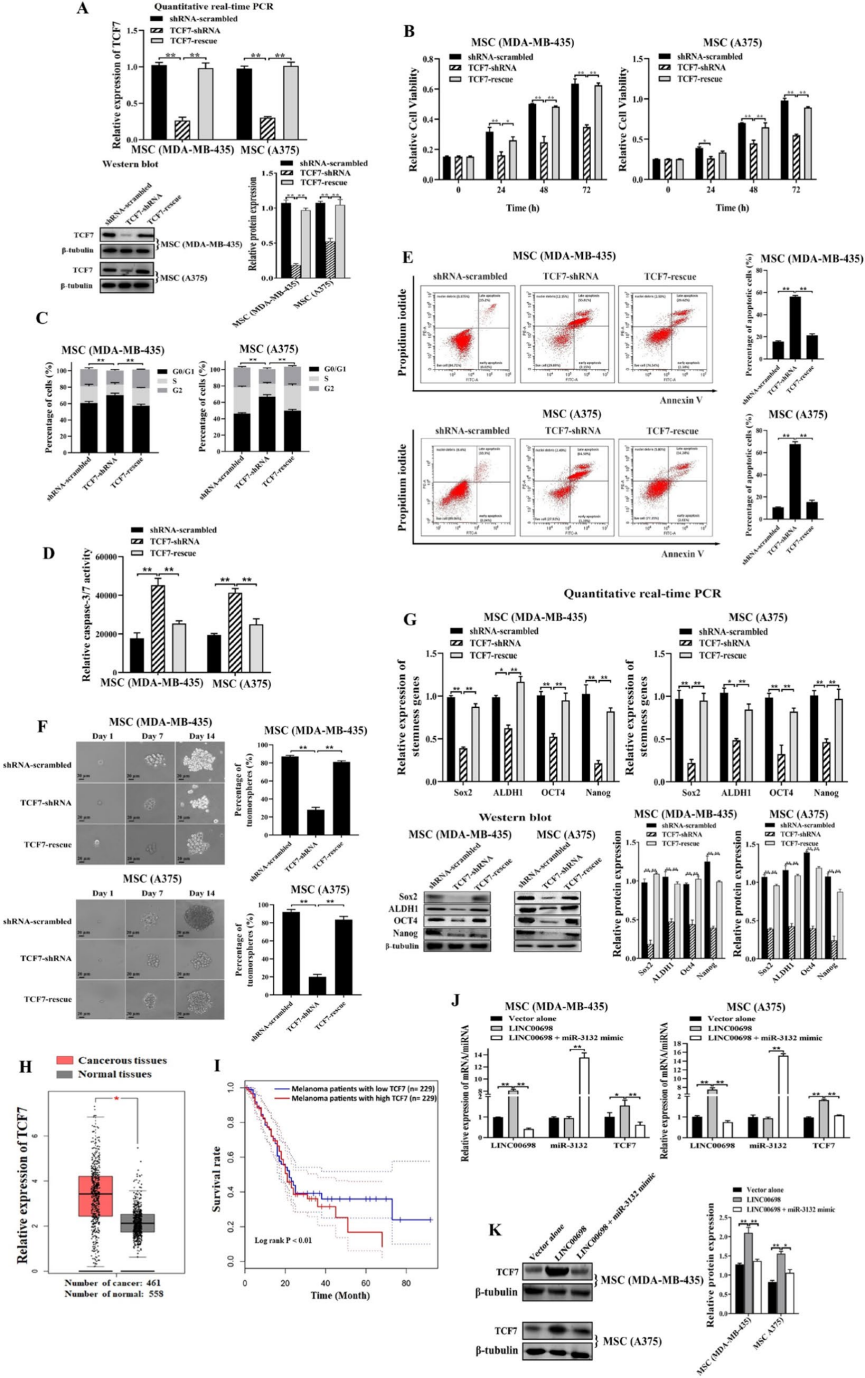
Requirement of TCF7 for MSCs. (**A**) Silencing or rescue of TCF7 in MSCs. MSCs were transfected with TCF7-shRNA to knock down the expression of the internal TCF7. As a control, shRNA-scrambled was included in the transfection. To rescue the expression of TCF7 in the TCF7-silenced cells, the cells were co-transfected with TCF7-shRNA and the plasmid expressing TCF7 (TCF7-rescue). The endogenous TCF7 was silenced by TCF7-shRNA in MSCs, while the plasmid expressed the recombinant TCF7 protein. At 48 h after transfection, TCF7 was examined using quantitative real-time PCR and Western blot (**, *p* < 0.01). β-tubulin was used as a control. (**B**) Impact of TCF7 knockdown and rescue on the proliferation of MSCs. The cell viability was examined at different times after transfection. ShRNA-scrambled was used as a control (*, *p* < 0.05; **, *p* < 0.01). (**C**) Role of TCF7 in cell cycle. The TCF7-silenced or rescued MSCs were subjected to the examination of cell cycle at 48 h after treatment (**, *p* < 0.01). (**D**) Influence of TCF7 knockdown and rescue on apoptosis of MSCs. The TCF7-silenced or rescued MSCs were subjected to caspase 3/7 activity assay at 48 h after transfection (**, *p* < 0.01). (**E**) Determination of apoptosis using Annexin V assays. Apoptosis of MSCs was detected by flow cytometry at 48 h after transfection with TCF7-shRNA, TCF7-rescue or shRNA-scrambled (**, *p* < 0.01). (**F**) Tumorsphere formation assay of TCF7-silenced or rescued MSCs. At 10 days after transfection, the percentage of tumorsphere formation of MSCs was evaluated (**, *p* < 0.01). Scale bar, 20 μm. (**G**) Effects of TCF7 on the expressions of stemness genes. The expressions of stemness genes in MSCs were examined using quantitative real-time PCR and Western blot at 48 h after the transfection (*, *p* < 0.05; **, *p* < 0.01). (**H**) Expression level of TCF7 in cancerous tissues of melanoma patients. According to the GEPIA database (http://gepia.cancer-pku.cn/index.html), the expression of TCF7 from melanoma patients in the cancerous tissues and normal tissues was estimated (*, *p* < 0.05). (**I**) Kaplan-Meier survival analysis. The Kaplan-Meier curve showed the survival curves of melanoma patients with low vs. high expression level of TCF7. (**J**) Expression level of LINC006987, miR-3132 and TCF7 in MSCs after transfection of vector alone, LINC00698 or LINC00698 and miR-3132 mimic. Quantitative real-time PCR was used to examine the expression levels of LINC006987, miR-3132 and TCF7 at 48 h after transfection (*, *p* < 0.05; **, *p* < 0.01). (**K**) Expression level of TCF7 detected by Western blot analysis. The TCF7 protein level in MSCs was detected at 48 h after transfection with vector alone, LINC00698 or LINC00698 and miR-3132 mimic (*, *p* < 0.05; **, *p* < 0.01)

To elucidate the role of TCF7 in MSCs, TCF7 was knocked down or rescued in MSCs, followed by the examination of the properties of MSCs. The results showed that TCF7 was silenced or rescued in MSCs (Fig. 4A). The TCF7 silencing led to a significant decrease of the viability of MSCs, while the cell viability of TCF7-rescued MSCs was comparable to the control (shRNA-scrambled) (Fig. 4B). These data indicated that TCF7 played a positive role in the proliferation of MSCs. The results demonstrated that the TCF7-silenced cells had a much higher percentage of cells in the G1 phase, and the TCF7 rescue in the TCF7-silenced MSCs restored the percentage of cells in the G1 phase that was comparable to the control (Fig. 4C), indicating that the TCF7 silencing led to cell cycle arrest in the G1 phase of MSCs. To illustrate whether cell cycle arrest resulted in apoptosis of MSCs, the apoptotic activity of TCF7-silenced or rescued MSCs was examined. The results showed that the TCF7 knockdown significantly enhanced the caspase 3/7 activity of MSCs compared to the control, while the caspase 3/7 activity in the TCF7-rescued MSCs was comparable to the control (Fig. 4D). Annexin V assays essentially yielded the same results (Fig. 4E). These results revealed that TCF7 could suppress apoptosis of MSCs.

To determine the impact of TCF7 on the stemness of MSCs, the capacity of tumorsphere formation of MSCs was evaluated. The results showed that the knockdown of TCF7 significantly reduced MSCs’ capacity to generate tumorspheres, while the percentage of tumorsphere formation of the TCF7-rescued MSCs was unchanged compared with the control (Fig. 4F). At the same time, the stemness genes were significantly downregulated in the TCF7-silenced MSCs and the TCF7 rescue recovered the expressions of stemness genes (Fig. 4G). These data indicated that TCF7 was required for the stemness of MSCs.

In clinic, TCF7 was significantly upregualted in the melanoma tissues compared to the normal tissues (Fig. 4H). The melanoma patients with higher TCF7 expression level had a worse survival rate than those with lower TCF7 expression level (Fig. 4I). These data indicated that TCF7 played a positive role in melanoma in clinic.

To confirm the influence of LINC00698-miR-3132-TCF7 axis on MSCs, MSCs were transfected with LINC00698, LINC00698 and miR-3132 mimic or vector alone, followed by the examination of LINC00698, miR-3132 and TCF7 expressions. The results revealed that the upregulated levels of TCF7 mRNA (Fig. 4J) and protein (Fig. 4K) resulted from LINC00698 overexpression which could be reversed by the miR-3132 overexpression (Fig. 4J and K), demonstrating the existence of the LINC00698-miR-3132-TCF7 axis in MSCs.

Taken together, these findings demonstrated that TCF7 was required for sustaining the stemness and the tumorigenic potency of MSCs.

### Influence of LINC00698-hnRNPM interaction on MSCs

**Fig. 5:**
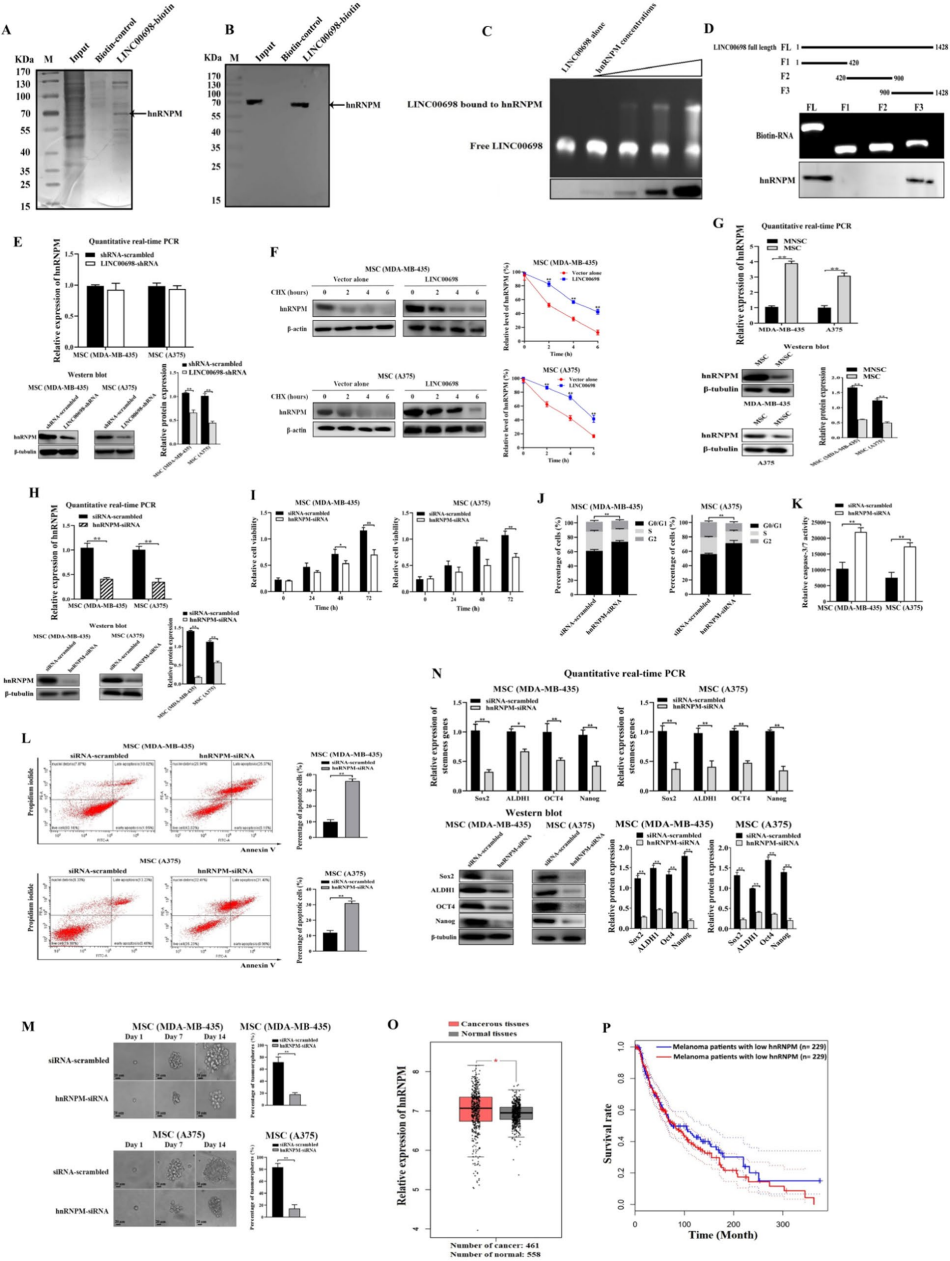
Influence of LINC00698-hnRNPM interaction on MSCs. (**A**) Identification of proteins bound to LINC00698. The lysate of MSCs was incubated with LINC00698-biotin beads to perform lncRNA pull-down assays. Biotin-control was used as a control. The proteins bound to LINC00698 were examined using SDS-PAGE with Coomassie blue staining. The arrow indicated the protein identified. M, protein marker. (**B**) Interaction between LINC00698 and hnRNPM protein. RNA pull-down assays using LINC00698 were performed. The pull-down products were subjected to Western blot using the hnRNPM-specific antibody. (**C**) Electrophoretic mobility shift assay (EMSA) of the LINC00698-hnRNPM interaction. LINC00698 was incubated with different concentrations of hnRNPM protein, followed by 1% agarose gel electrophoresis. As a control, LINC00698 RNA alone was included in the assays. (**D**) The fragment of LINC00698 bound to hnRNPM protein. Based on the secondary structure, LINC00698 was truncated (up). The in vitro transcribed biotin-labeled RNAs were analyzed with 1% agarose gel electrophoresis (middle). The biotin-labeled RNAs were incubated with the lysate of MSCs and then the proteins bound to RNAs were subjected to Western blot using the hnRNPM-specific antibody (bottom). (**E**) Influence of LINC00698 on the stability of hnRNPM protein in MSCs. The LINC00698-silenced MSCs at 48 h after LINC00698-shRNA transfection were subjected to quantitative real-time PCR and Western blot to detect the hnRNPM mRNA and protein, respectively. As a control, shRNA-scrambled transfection was included in the assays (**, *p* < 0.01). In Western blot, β-tubulin was used as a control. (**F**) Western blot to assess the impact of LINC00698 on the hnRNPM protein stability. To evaluate the influence of LINC00698 on the stability of hnRNPM protein, the cycloheximide chasing assay was performed. MSCs (MDA-MB-435 and A375) were transfected with a vector expressing LINC00698 or vector alone and then treated with 50 µg/mL cycloheximide (CHX). At different time after treatment (0, 2, 4 and 6 h), the cells were analyzed with Western blot. β-actin was used as an internal control (**, *p* < 0.01). (**G**) Differential expression of hnRNPM in MSCs and MNSCs. The expression level of hnRNPM was detected using quantitative real-time PCR and Western blot (**, *p* < 0.01). β-tubulin was used as a control. (**H**) Silencing of hnRNPM in MSCs. MSCs (MDA-MB-435 and A375) were transfected with hnRNPM-siRNA or a control siRNA-scrambled. At 48 h after transfection, the expression of hnRNPM was evaluated using quantitative real-time and Western blot (**, *p* < 0.01). β-tubulin was used as a control. (**I**) Impact of hnRNPM silencing on the proliferation of MSCs. MSCs transfected with hnRNPM-siRNA or siRNA-scrambled were subjected to cell viability assays at different time after transfection. (*, *p* < 0.05; **, *p* < 0.01). (**J**) Effects of hnRNPM silencing on cell cycle. The cell cycle of hnRNPM-siRNA or siRNA-scrambled-transfected MSCs was assessed using flow cytometry at 48 h after transfection (**, *p* < 0.01). (**K**) Impact of hnRNPM silencing on caspase 3/7 activity of MSCs. The caspase 3/7 activity of MSCs transfected with hnRNPM-siRNA or siRNA-scrambled was analyzed at 48 h after transfection (**, *p* < 0.01). (**L**) Role of hnRNPM in apoptosis of MSCs. MSCs transfected with hnRNPM-siRNA or siRNA-scrambled were subjected to annexin V assays at 48 h after transfection (**, *p* < 0.01). (**M**) Tumorsphere formation assay of the hnRNPM-silenced MSCs. The hnRNPM-silenced MSCs were subjected to tumorsphere formation assays. As a control, siRNA-scrambled was included in the assays. At 10 days after transfection, the percentage of tumorsphere formation was evaluated (**, *p* < 0.01). Scale bar, 20 μm. (**N**) Effects of hnRNPM silencing on the stemness genes’ expressions of MSCs. The expressions of stemness genes in MSCs transfected with hnRNPM-siRNA or siRNA-scrambled were examined using quantitative real-time PCR and Western blot (*, *p* < 0.05; **, *p* < 0.01) at 48 h after transfection. (**O**) Relative expression level of hnRNPM in cancerous tissues of melanoma patients. According to the GEPIA database, the expression level of hnRNPM in the cancerous tissues and normal tissues of melanoma patients was evaluated (*, *p* < 0.05). (**P**) Kaplan-Meier survival analysis. The Kaplan-Meier curve showed the survival rate of melanoma patients with low vs. high expression level of hnRNPM

To reveal the proteins interacted with LINC00698, RNA pull-down assays were performed. The results illustrated a protein was specifically interacted with LINC00698 in MSCs (Fig. 5A). The mass spectrometric data identified that this protein was hnRNPM (Fig S2). Western blot analysis confirmed the interaction between LINC00698 and hnRNPM protein (Fig. 5B).

To further explore the interaction between LINC00698 and hnRNPM, electrophoretic mobility shift assay (EMSA) was performed. The results showed that the LINC00698-hnRNPM complex was shifted compared with LINC00698 alone (Fig. 5C). These data indicated that LINC00698 tremendously bounds to hnRNPM in MSCs.

To determine the fragment of LINC00698 interacted with the hnRNPM protein, LINC00698 was truncated based on the secondary structure of LINC00698 and then the biotin-labeled LINC00698 pulldown assay was performed. The results showed that the 3’-end of LINC00698 (nucleotide 900 to 1428) was bound to the hnRNPM protein (Fig. 5D). These findings revealed that LINC00698 preferentially interacted with the hnRNPM protein at its 3ˊ-end.

To assess the impact of LINC00698 on the stability of the hnRNPM protein, the mRNA and protein levels of hnRNPM in the LINC00698-silenced MSCs were examined. The results indicated that there was no significant difference of the hnRNPM mRNA level between LINC00698 knockdown and the control (Fig. 5E). However, the hnRNPM protein was significantly reduced in the LINC00698-silenced MSCs compared with the control (Fig. 5E). To further confirm the impact of LINC00698 on the stability of hnRNPM protein, the LINC00698-overexpressed MSCs were treated with cycloheximide (CHX), a protein synthesis inhibitor. The results revealed that the hnRNPM protein was more stable in the LINC00698-overexpressed MSCs than the control (Fig. 5F). These data demonstrated that the binding of LINC00698 to the hnRNPM protein enhanced the stability of hnRNPM protein.

To reveal the role of hnRNPM in MSCs, the expression of hnRNPM was examined in MSCs and MNSCs. The data of quantitative real-time PCR and Western blot indicated that hnRNPM was significantly upregulated in MSCs (Fig. 5G), suggesting an important role of hnRNPM in MSCs.

To investigate the influence of hnRNPM on MSCs, hnRNPM was knocked down in MSCs, followed by the examination of cell properties. The results of quantitative real-time PCR and Western blot showed that hnRNPM was silenced in MSCs by hnRNPM-siRNA (Fig. 5H). The hnRNPM silencing caused a significant decrease of MSCs’ viability compared to the control (Fig. 5I) and cell cycle arrest in G0/G1 phase (Fig. 5J), indicating a favorable function of hnRNPM in the proliferation of MSCs. The cell cycle arrest in G0/G1 phase led to apoptosis of MSCs (Fig. 5K and L).

To assess the role of hnRNPM in the stemness of MSCs, the tumorsphere formation capability of the hnRNPM-silenced MSCs was examined. The results showed that the hnRNPM depletion significantly decreased the ability of MSCs to form tumorspheres compared with the control (Fig. 5M). At the same time, the stemness genes were significantly reduced in the hnRNPM-silenced MSCs (Fig. 5N). These results presented that hnRNPM played a crucial role in the stemness of MSCs.

To figure out the impact of hnRNPM on melanoma in clinic, the expression level of hnRNPM in melanoma patients was analyzed. The results displayed that hnRNPM was significantly upregulated in the cancerous tissues of melanoma patients compared to that of the normal tissues (Fig. 5O). At the same time, the individuals with higher hnRNPM expression level had worse overall survival rate compared to those with lower hnRNPM expression level (Fig. 5P). These data indicated that hnRNPM played a positive role in melanoma in clinic.

Collectively, these data revealed that LINC00698 could bind to the hnRNPM protein to enhance the protein stability, thus suppressing apoptosis and promoting the stemness of MSCs.

### Role of LINC00698-miR-3132-TCF7/hnRNPM axis in MSCs

**Fig. 6:**
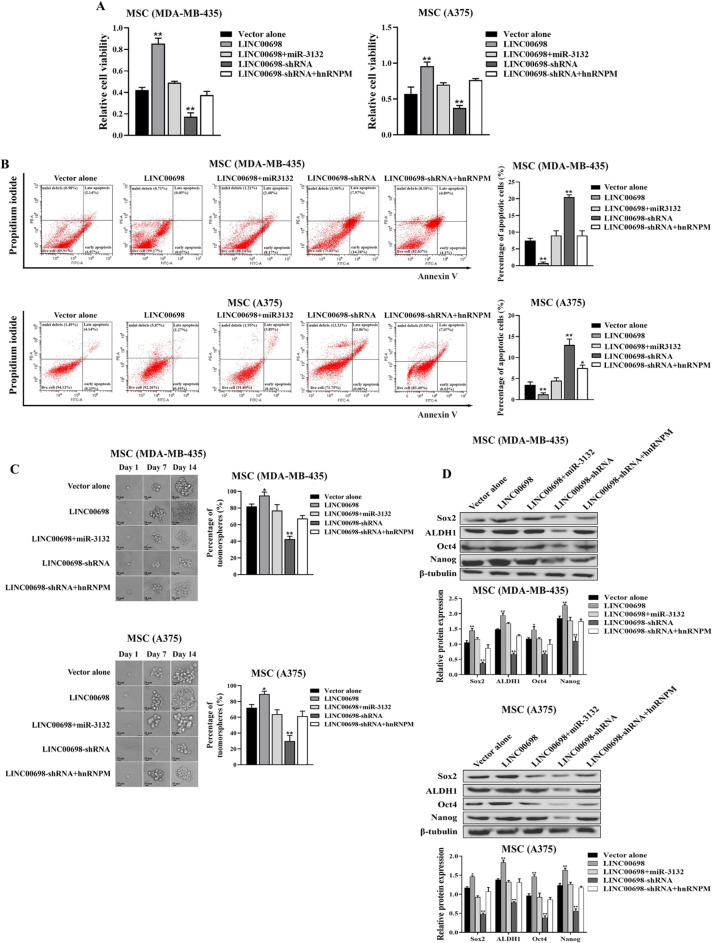
Role of LINC00698-miR-3132-TCF7/hnRNPM axis in MSCs. (**A**) Effects of LINC00698-miR-3132-TCF7/hnRNPM axis on the proliferation of MSCs. The cell viability was examined at 48 h after transfection with LINC00698, LINC00698 and miR-3132 mimic, LINC00698-shRNA, LINC00698-shRNA and hnRNPM overexpression or vector alone (**, *p <* 0.01). (**B**) Examination of apoptosis using Annexin V assays. Apoptosis of MSCs was detected by flow cytometry at 48 h after treatments (*, *p <* 0.05; **, *p <* 0.01). (**C**) Tumorsphere formation assay of MSCs. At 10 days after transfection with LINC00698, LINC00698 and miR-3132 mimic, LINC00698-shRNA, LINC00698-shRNA and hnRNPM or vector alone, the percentage of tumorsphere formation of MSCs was evaluated (*, *p <* 0.05; **, *p <* 0.01). Scale bar, 20 μm. (**D**) Impact of LINC00698-miR-3132-TCF7/hnRNPM axis on the expressions of stemness genes. The expressions of stemness genes in MSCs were examined using Western blot at 48 h after transfection (*, *p <* 0.05; **, *p <* 0.01)

To evaluate the regulatory role of the LINC00698-miR-3132-TCF7/hnRNPM axis in the proliferation and stemness of MSCs, the functional rescue experiments were conducted. MSCs were transfected with LINC00698, LINC00698 and miR-3132 mimic, LINC00698-shRNA, LINC00698-shRNA and hnRNPM overexpression vector or vector alone, followed by the examination of MSC properties. The results demonstrated that miR-3132 and hnRNPM protein functionally reversed the proliferation and apoptosis effects induced by the LINC00698 overexpression or silencing in MSCs, respectively (Fig. 6A and B). Furthermore, miR-3132 reversed the effects of the LINC00698 overexpression on the stemness of MSCs, while hnRNPM reversed the stemness induced by the LINC00698 silencing in MSCs (Fig. 6C and D).

Collectively, these data revealed the functionally regulatory role of the LINC00698-miR-3132-TCF7/hnRNPM axis in MSCs, which promoted the proliferation and stemness of MSCs and suppressed apoptosis of MSCs.

### Role of LINC00698 on tumorigenesis of MSCs in vivo

**Fig. 7:**
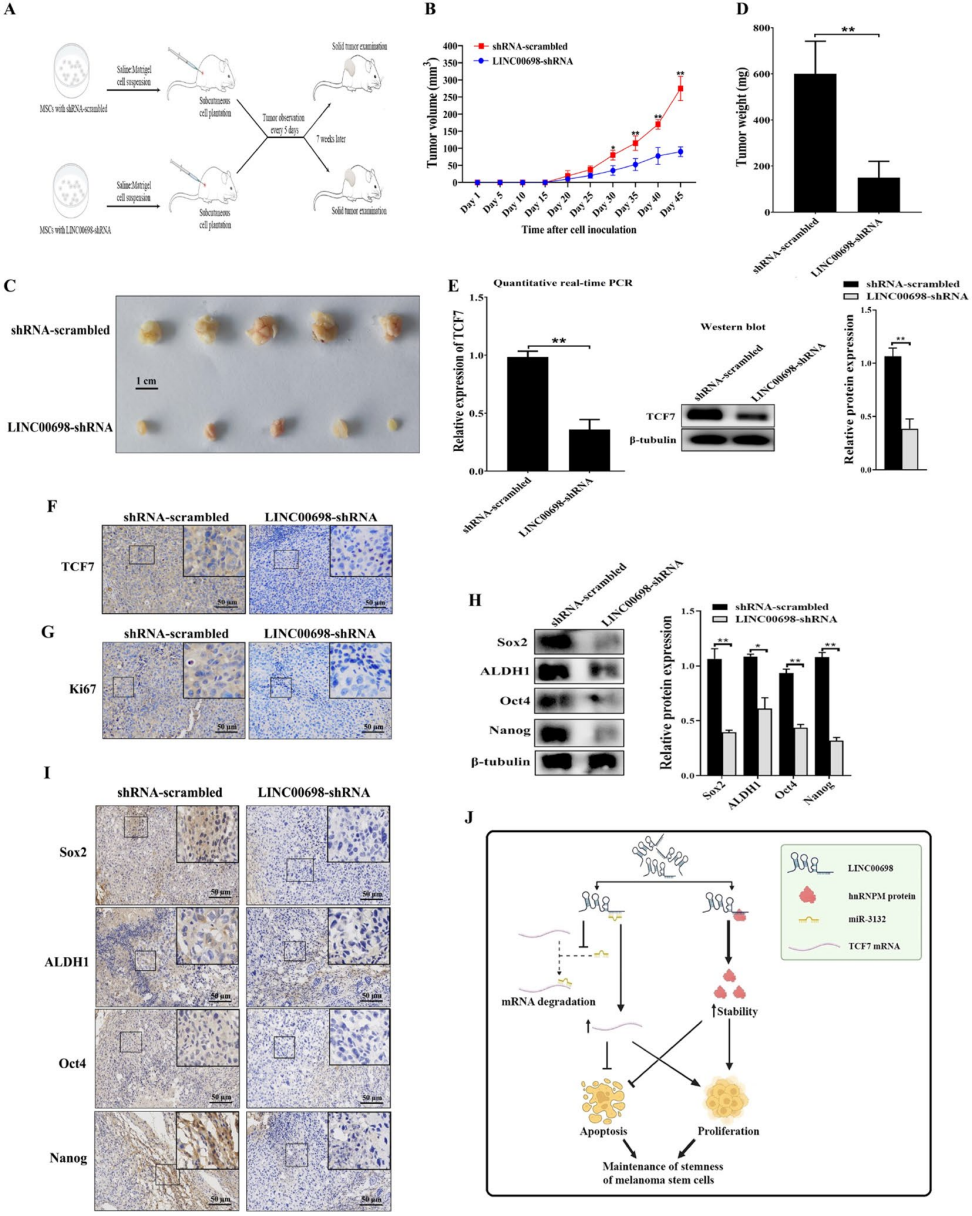
Role of LINC00698 on tumorigenesis of MSCs in vivo. (**A**) Schematic diagram of the in vivo experiments. MSCs stably expressing LINC00698-shRNA or shRNA-scrambled were inoculated into non-obese diabetes/severe combined immunodeficiency (NOD/SCID) mice. The tumor volume was examined every five days and the mice were sacrificed at week 7. (**B**) Influence of LINC00698 silencing on the tumor growth in mice. The tumor volume in mice was examined every 5 days. The value of each point represented the mean tumor volume of 5 mice (*, *p* < 0.05; **, *p* < 0.01). (**C**) Assessment of the solid tumor size excised from mice. The mice were euthanized seven weeks after cell inoculation, and the solid tumor size was evaluated. (**D**) Effects of LINC00698 silencing on the solid tumor weight. The data represented the tumor weight of 5 mice for each treatment (**, *p* < 0.01). (**E**) Evaluation of TCF7 expression level in the solid tumors of mice. Quantitative real-time PCR (**, *p* < 0.01) and Western blot were used to detect the TCF7 expression. β-tubulin was used as a control. (**F**) Immunohistochemical analysis of TCF7 protein in the solid tumor of mice. Brown represented the TCF7 protein and blue indicated the nuclei stained by hematoxylin. Scale bar, 50 μm. (**G**) Immunohistochemical analysis of Ki67 protein in the solid tumors of mice. Brown indicated the Ki67 protein and blue signified nuclei stained by hematoxylin. Scale bar, 50 μm. (**H**) Evaluation of the expressions of stemness genes in the solid tumors of mice. Western blot was used to detect the stemness gene expression levels in the solid tumors of the mice treated with LINC00698-shRNA or shRNA-scrambled. β-tubulin was used as a control (*, *p* < 0.05; **, *p* < 0.01). (**I**) Immunohistochemical analysis of the proteins of stemness genes in the solid tumor of mice. Brown showed the Sox, ALDH1, Oct4 or Nanog protein. Blue indicated the nuclei stained by hematoxylin. Scale bar, 50 μm. (**J**) Model for the underlying mechanism of LINC00698 in tumorigenesis of MSCs

To determine the influence of LINC00698 on tumorigenesis of MSCs in vivo, the LINC00698-silenced MSCs were injected into non-obese diabetes/severe combined immunodeficiency (NOD/SCID) mice, followed by the examination of tumors (Fig. 7A). The date of tumor growth curve analysis showed that the LINC00698 silencing significantly suppressed the tumor growth in mice compared with the control (Fig. 7B). The tumor sizes of xenografted solid tumors in the LINC00698-silenced mice were considerably smaller than those of the control mice (Fig. 7C). The weights of the solid tumors in the LINC00698-silenced mice were significantly decreased compared with the controls (Fig. 7D).

To explore whether LINC00698 functioned via the LINC00698-miR-3132-TCF7 axis in vivo, the expression level of TCF7 in the solid tumors of the mice transfected with the LINC00698-silenced MSCs was examined. The results demonstrated that the TCF7 expression was significantly reduced in the mice transfected with the LINC00698-silenced MSCs compared with that in the control mice (Fig. 7E). Immonohistochemical analysis yielded essentiallly the similar results (Fig. 7F). These data revealed that LINC00698 could suppress tumorigeneis of MSCs in vivo via the LINC00698-miR-3132-TCF7 pathway.

To assess the impact of LINC00698 on the proliferation of MSCs in vivo, the expression level of ki67, a marker of cell proliferation, in the solid tumors of mice was examined. The results of immunohistochemical analysis showed that the Ki67 protein level was significantly reduced in the solid tumors of the LINC00698-silenced mice compared to that in the control mice (Fig. 7G), indicating that LINC00698 promoted the cell proliferation in mice.

To determine the impact of LINC00698 on the stemness of MSCs in vivo, the expression levels of the stemness genes in the solid tumors of mice were examined. The results of Western blot (Fig. 7H) and immunohistochemistry data (Fig. 7I) showed that the protein levels of stemness genes were significantly reduced in the solid tumors of LINC00698-silenced mice compared to those in the control mice. These results demonstrated that LINC00698 promoted the stemness of MSCs stemness in mice.

Taken together, these findings revealed that LINC00698 was essential for the maintenance of MSCs’ stemness via sponging miR-3132 to inhibit the mRNA degradation of TCF7 mediated by miR-3132 and directly interacting with the hnRNPM protein to enhance the hnRNPM stability (Fig. 7J). The interactions between LINC00698 and miR-3132 as well as LINC00698 and the hnRNPM protein promoted the proliferation and suppressed apoptosis of MSCs (Fig. 7J).

## Discussion

Melanoma, the most lethal kind of skin cancer in humans, has attracted much attention in recent years [1, 30]. Despite the existing possibilities for melanoma therapy, the recurrence rate remains high and only a few patients gain long-term survival [31]. Because of its capacity to develop resistance to multiple treatment regimens, metastatic melanoma is a cancer that is famously difficult to cure [32]. Therefore, a deeper comprehension of the biology of melanoma and potential factors underlying poor patient response to therapies is urgently required. As crucial regulators of modulating genome-wide fluctuations in gene transcriptional activity, lncRNAs have attracted more and more pieces of attention [33]. In the preceding decade, novel insights into the causes, mechanisms, and potential therapies for various clinical disorders, including cancers, have been revealed in lncRNAs [34]. As reported, lncRNAs can govern the progression of various malignancies, as oncogenes or tumor suppressors, by interactions with proteins or nucleic acids [35]. Nevertheless, the role of lncRNAs in cancer stem cells has not been sufficiently investigated. In this study, the results demonstrated that LINC00698 was required for tumorigenesis of MSCs via the suppression of apoptosis and the maintenance of the stemness of MSCs. Therefore, our study presented a new lncRNA which was responsible for regulating the stemness of MSCs.

In this study, our findings demonstrated that LINC00698 was required for maintaining the stemness of MSCs through targeting miR-3132 to upregulate TCF7 and binding to the hnRNPM protein to enhance the protein stability, thus leading to the suppression of apoptosis and the promotion of proliferation of MSCs. TCF7, a member of T cell factor (TCF) family, functions as a transcriptional activator [36]. Numerous studies have provided significant evidence to indicate that the upregulation of TCF7 is associated with progression or worse prognosis in various types of cancers [37–40]. The TCF family plays important roles in the Wnt signalling pathway by interacting with translocated β-catenin and facilitating the transcription of target genes [41, 42]. The fundamental role of the Wnt signaling pathway involves cellular self-renewal and proliferation [43]. Some investigations have identified that TCF7 can play a vital role in tumorigenesis of cancer stem cells [44, 45]. In our study, the *in vitr*o and in vivo data presented that the LINC00698-miR-3132-TCF7 axis was required to maintain the stemness and the tumorigenicity of MSCs. Thus, our findings provided novel perspectives on the role of LINC00698-mediated tumorigenesis of MSCs. Except for the LINC00698-miR-3132-TCF7 axis, our study found that LINC00698 could bind to the hnRNPM protein to increase the protein stability in MSCs and hnRNPM was required for the stemness of MSCs. The in vivo LINC00698-miR-3132 and the LINC00698-hnRNPM interactions could be further explored in the future. As reported, the hnRNPM protein is an essential splicing factor that functions as a vital component in multiple cases of cancer metastasis, such as breast cancer [46], gastric cancer [47], colon cancer [48], hepatocellular carcinoma [49] and prostate cancer [50]. In recent years, the increasing evidence confirms that the hnRNPM protein plays a crucial role in regulating cancer stem cells properties [51, 52]. In this context, our findings offered novel insights into the involvement of lncRNAs in tumorigenesis of MSCs by elucidating the interaction with the hnRNPM protein. Collectively, our study contributed a comprehensive understanding of a newly identified mechanism that governed the functional interaction between LINC00698 and miR-3132 as well as LINC00698 and the hnRNPM protein in MSCs. In the future, the roles of LINC00698 as well as the LINC00698-mediated pathway in metastasis of MSCs would be further investigated.

### Electronic supplementary material

Below is the link to the electronic supplementary material.

Supplementary Material 1

Supplementary Material 2

## Data Availability

No datasets were generated or analysed during the current study.
