# Supplementary material for: Oncogenic LINC00698 suppresses apoptosis of melanoma stem cells to promote tumorigenesis via LINC00698-miR-3132-TCF7/hnRNPM axis
Source: Cancer Cell Int. 2024 Jul 28;24:269. doi: 10.1186/s12935-024-03408-z (PMC11283696; doi:10.1186/s12935-024-03408-z)

Supplementary figures

Figure legends

Fig S1. Relative expression of three differentially expressed lncRNAs in various cancers. The data of the expression levels of LINC01198, ATP13A4-AS1 and EPHA5-AS1 in different cancers were obtained from the MiTranscriptome database.

Fig S2. Identification of hnRNPM protein by mass spectrometry. The hnRNPM unique peptides were identified by mass spectrometry analysis. The matched peptides were underlined.

Fig S1

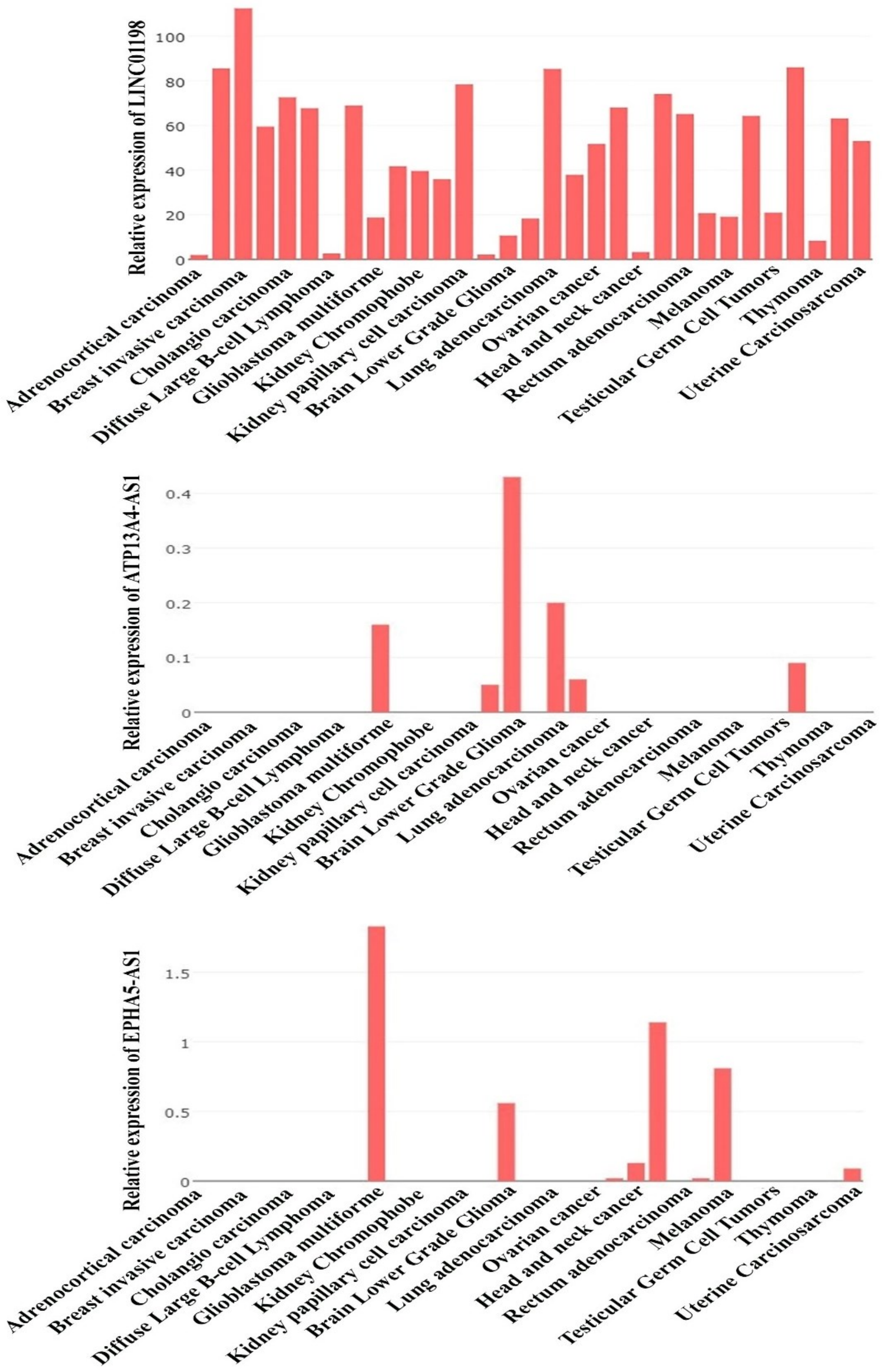

Relative expression of LINC01198
100
80
60
40
20
0
Adrenocortical carcinoma
Breast invasive carcinoma
Cholangio carcinoma
Diffuse Large B-cell Lymphoma
Glioblastoma multiforme
Kidney Chromophobe
Kidney papillary cell carcinoma
Brain Lower Grade Glioma
Lung adenocarcinoma
Ovarian cancer
Head and neck cancer
Rectum adenocarcinoma
Melanoma
Testicular Germ Cell Tumors
Thymoma
Uterine Carcinosarcoma
Relative expression of ATP13A4-AS1
0.4
0.3
0.2
0.1
0
Adrenocortical carcinoma
Breast invasive carcinoma
Cholangio carcinoma
Diffuse Large B-cell Lymphoma
Glioblastoma multiforme
Kidney Chromophobe
Kidney papillary cell carcinoma
Brain Lower Grade Glioma
Lung adenocarcinoma
Ovarian cancer
Head and neck cancer
Rectum adenocarcinoma
Melanoma
Testicular Germ Cell Tumors
Thymoma
Uterine Carcinosarcoma
Relative expression of EPHA5-AS1
1.5
1
0.5
0
Adrenocortical carcinoma
Breast invasive carcinoma
Cholangio carcinoma
Diffuse Large B-cell Lymphoma
Glioblastoma multiforme
Kidney Chromophobe
Kidney papillary cell carcinoma
Brain Lower Grade Glioma
Lung adenocarcinoma
Ovarian cancer
Head and neck cancer
Rectum adenocarcinoma
Melanoma
Testicular Germ Cell Tumors
Thymoma
Uterine Carcinosarcoma

Fig S2

hnRNPM protein

MAAGVEAAAEVAATEIK**MEEESGAPGVPSGNGAPGPK**GEGERPAQNEKRKEKNIKRGGNR
FEPYANPTKRYR**AFITNIPFDVKWQSLKDLVKEKVGEVTYVELLMDAEGK**SRGCAVVEFK
MEESMKKAAEVLNKHSLSGRPLKVKEDPDGEHARRAMQK**VMATTGGMGMGPGGPGMITIP**
PSILNNPNIPNEIIHALQAGRLGSTVFVANLDYKVGWKKLKEVFSMAGVVVR**ADILEDKD**
GKSRGIGTVTFEQSIEAVQAISMFNGQLLFDRPMHVKMDERALPK**GDFFPPERPQQLPHG**
LGGIGMGLGPGGQPIDANHLNKGIGMGNIGPAGMGMEGIGFGINKMGGMEGPFGGGMENM
GRFGSGMNMGRINEILSNALKRGEIIAKQGGGGGGGSVPGIERMGPGIDRLGGAGMERMG
AGLGHGMDRVGSEIERMGLVMDRMGSVERM**GSGIERMGPLGLDHMASSIERMGQT**MERIG
SGVERMGAGM**GFGLERMAAPIDRVGQTIERMGSGVERMGP**AIERMGLSMERMVPAGMGAG
LERMGPVMDRMATGLERMGANNLERMGLERMGANSLERMGLERMGANSLERMGPAMGPAL
GAGIERMGLAMGGGGGASFDRAIEMER**GNFGGSFAGSFGGAGGHAPGVAR**KACQIFVRNL
PFDFTWKMLK**DKFNECGHVLYADIK**MENGK**SKGCGVVKFESPEVAER**ACR

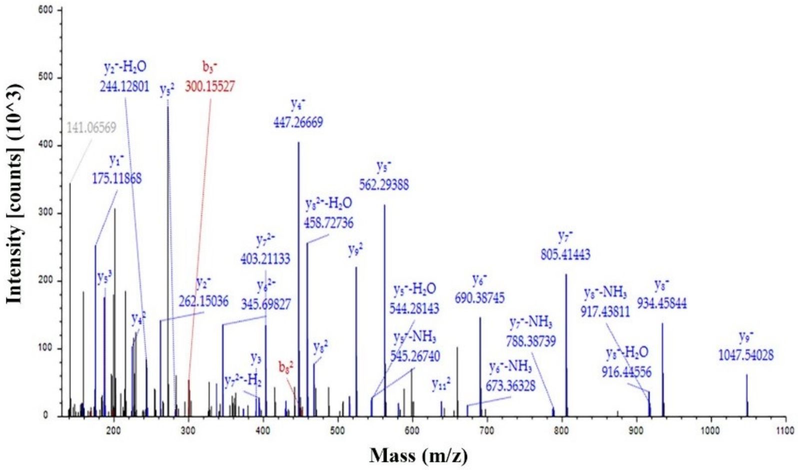

Supplement: Supplementary file 1 — Supplementary Material 1 [file 12935_2024_3408_MOESM1_ESM.pdf]
